# Identification of Mycobacterium pseudoshottsii in the Eastern Mediterranean

**DOI:** 10.1128/spectrum.00856-23

**Published:** 2023-06-05

**Authors:** Nadav Davidovich, Andrei Makhon, Gal Zizelski Valenci, Zeev Dveyrin, Tal Yahav, Tobia Pretto, Dan Tchernov, Danny Morick

**Affiliations:** a Morris Kahn Marine Research Station, University of Haifa, Haifa, Israel; b Israeli Veterinary Services, Bet Dagan, Israel; c National Public Health Laboratory, Public Health Directorate, Ministry of Health, Tel Aviv, Israel; d Bioinformatics Services Unit, University of Haifa, Haifa, Israel; e Istituto Zooprofilattico Sperimentale delle Venezie, Legnaro, Padua, Italy; f Hong Kong Branch of Southern Marine Science and Engineering Guangdong Laboratory (Guangzhou), Hong Kong, China; University of Pittsburgh School of Medicine

**Keywords:** *Mycobacterium pseudoshottsii*, nontuberculous mycobacteria, Israel, emerging pathogen

## Abstract

Among the numerous pathogenic nontuberculous mycobacteria (NTM), which may cause disease in both poikilothermic and homoeothermic organisms, members of the unique clade Mycobacterium ulcerans/Mycobacterium marinum (MuMC) may cause disease in both fish and humans. Here, we describe the emergence of Mycobacterium pseudoshottsii, one of the four MuMC members, in Israel. For many years, M. marinum was the dominant NTM that was diagnosed in Israel as a fish pathogen. To the best of our knowledge, this is the first isolation and genomic characterization of *M. pseudoshottsii* infecting edible fish from two different fish species farmed in offshore sea cages in the eastern Mediterranean as well as in a recirculating aquaculture system in Israel. We compared the *M. pseudoshottsii* whole-genome sequences to all available genomic sequences of MuMC in free, publicly accessible databases.

**IMPORTANCE** Mycobacterium pseudoshottsii was first detected in 1997 in the USA, infecting wild striped bass (Morone saxatilis). Since then, several reports from different countries worldwide have shown its capacity to become established in new regions as well as its pathogenicity to saltwater and euryhaline finfish of different genera. Our phylogenetic analysis revealed that the Mycobacterium ulcerans/Mycobacterium marinum clade (MuMC) is divided into two main branches: one that includes M. marinum and *M. pseudoshottsii*, and the second, which includes other M. marinum isolates as well as two isolates of M. shottsii. Our results reinforce the proposition that the geographical distribution of *M. pseudoshottsii* is much more extensive than is commonly believed. The emergence of *M. pseudoshottsii* in different parts of the world and its pathogenic traits that affect finfish of different genera may be a cause for concern among fish farmers, researchers, and environmental organizations.

## OBSERVATION

The nontuberculous mycobacteria (NTM) are a large group of pathogens that are capable of causing chronic and severe disease in a vast number of living organisms (1). The Mycobacterium ulcerans/Mycobacterium marinum clade (MuMC) is an important group of NTM that shows high genomic similarities among its four members: Mycobacterium ulcerans, which causes a necrotizing disease of the skin and soft tissue and is also known as Buruli ulcer, especially in African countries and Australia (2); Mycobacterium marinum, which is one of the most important fish pathogens but is also known as a human pathogen (3); Mycobacterium shottsii, which is a fish-pathogenic mycobacterium that was originally isolated from wild, diseased striped bass (Morone saxatilis) in the Chesapeake Bay, USA, with a limited geographical distribution (4); and Mycobacterium pseudoshottsii, which is a fish pathogen that was also first detected in wild, diseased striped bass in the Chesapeake Bay (5) but, with time, was also diagnosed in farmed fish species from Japan (6) and Europe (7).

From 2020 to 2021, public veterinarians working at two Israeli fish-sorting stations noted splenomegaly and pathological alterations of internal parenchymal organs that were referable to mycobacteriosis in 5 to 10% of examined specimens during a routine visual inspection as part of a specified organoleptic examination for the premarketing control of locally grown, edible fish (8). We collected fish tissue samples from both farms: (i) a small-scale recirculating aquaculture system (RAS) that was growing hybrid striped bass (*Morone chrysops* × *M. saxatilis*) and (ii) an offshore sea cage farm in the eastern Mediterranean Sea that was rearing gilthead seabream (Sparus aurata). Our epidemiological investigation revealed that both farms had purchased fingerlings from the same hatchery. The source farm also has breeding herds of both white bass and striped bass that were originally imported from the USA, where *M. pseudoshottsii* is endemic. Hitherto, M. marinum had been the only infectious agent that was causing piscine mycobacteriosis in Israel (3), and this species was immediately suspected. Samples of fish parenchymal organs (liver, heart, kidney, gills, visceral adipose tissue, and intestine) were smeared on glass slides and subjected to Ziehl-Neelsen (ZN) staining (9). This yielded rod-shaped bacteria that were referable to the genus Mycobacterium. The fish specimens were further fixed in 10% buffered formalin for 48 h, dehydrated in an ethanol series and xylene, and embedded in Paraplast. Standard histological protocols were applied. Finally, the samples were cut into 3 μm sections and alternately stained with Mayer’s hematoxylin and eosin (H&E), histochemical ZN stain, and Gram-Twort stain.

**FIG 1:**
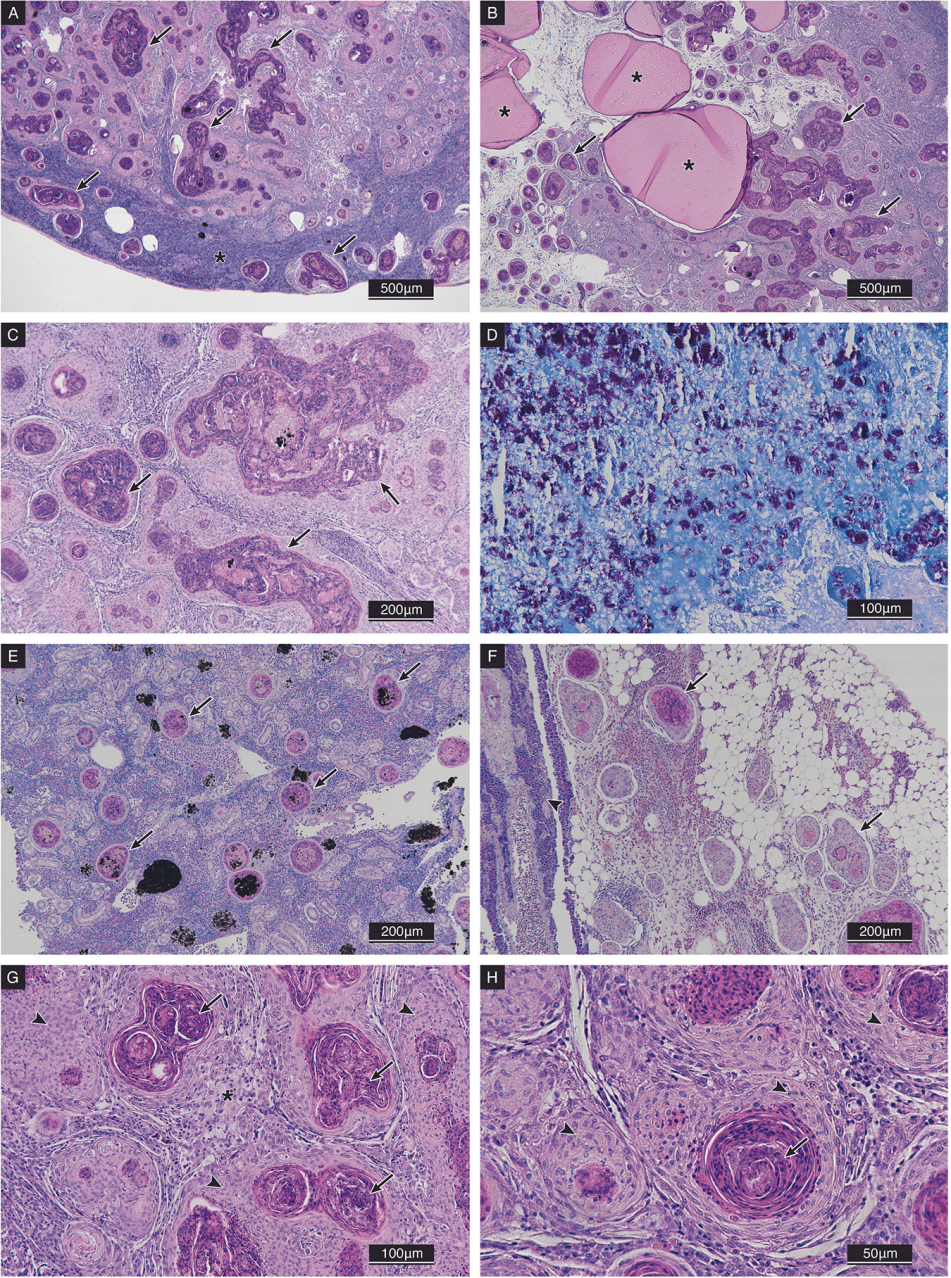
Histological alterations observed in gilthead seabream affected by Mycobacterium pseudoshottsii. (A) Spleen parenchyma showing severe multifocal to coalescing granulomatous inflammation (arrows), peripheral normal spleen parenchyma (*), H&E, 4×. (B) Affected spleen core showing multiple amorphous eosinophilic areas (*) that are surrounded by multifocal granulomatous inflammation (arrows), H&E, 4×. (C) Detail of the granulomatous reaction (arrows) in the spleen, H&E, 10×. (D) Diffuse presence of acid-fast bacilli that were referable to the genus Mycobacterium and were interspersed between the amorphous matrix in the spleen core, ZN, 20×. (E) Multifocal granulomas (arrows) in trunk kidney parenchyma, H&E, 10×. (F) Multifocal granulomas (arrows) in the visceral adipose tissue and the pancreas (arrowhead), H&E, 10×. (G) Detail of coalescent epithelioid cell granulomas (arrowheads) with a necrotic core (arrows) and vacuolated macrophages (*) in the spleen, H&E, 20×. (H) Increased magnification of the epithelioid cell granulomas (arrowheads) with an eosinophilic necrotic core (arrows) H&E, 40×.

Histopathology (Fig. 1) revealed the marked presence of multifocal to coalescing granulomas in the spleen as well as amorphous eosinophilic areas in the spleen parenchyma with an intense infiltration of acid-fast bacilli (AFB) that were referable to Mycobacterium spp. These AFB were evident in the granuloma cores in the liver, heart, kidney, visceral adipose tissue, and intestinal serosa, and they were also dispersed in the connective tissue.

The tissue samples of the fish with splenomegaly or splenic granulomatous nodules were also crushed in a mortar with a few drops of deuterium-depleted water (DDW) and decontaminated for 30 min at room temperature with a 1:1 volume of 5% oxalic acid. Then, DDW was added to stop the process, and the sample was centrifuged at 3,000 × *g* for 20 min and resuspended in 5 mL of the supernatant. These samples were inoculated on solid Löwenstein-Jensen (LJ) medium and incubated at 25°C and 30°C until growth was observed. Overall, one isolate was obtained from hybrid striped bass (reference no. 189-2020) that were incubated at 25°C, and two isolates were obtained from gilthead seabream (reference no. 539-2021, 540-2021) that were incubated at 25°C. The first bacterial colonies were observed on LJ medium for samples 189-2020, 539-2021, and 540-2021 after 29, 21, and 25 days, respectively. The smears of all of the mycobacterial colonies were subjected to ZN staining (9). Interestingly, no growth of mycobacterial colonies was observed under incubation at 30°C, as was noted with a previous case of piscine mycobacteriosis that was caused by M. marinum affecting gilthead seabream in Israel (3). These *in vitro* thermal growth characteristics matched those of a recently published study in which elevated temperature inhibited *M. shottsii* infection and *M. pseudoshottsii* disease *in vivo* (10).

For the whole-genome sequencing, 3 representative colonies were collected from LJ medium using an inoculating loop and were suspended in 2 mL of DDW. DNA was then extracted from the cultures by using a QIAamp DNA Mini Kit (Qiagen), according to the manufacturer’s protocol. The genomic DNA of the Mycobacterium 189-2020, 539-2021 and 540-2021 DNA libraries was prepared by using a Nextera DNA Flex Library Preparation Kit, following the manufacturer’s instructions. Sequencing was performed on an Illumina MiSeq platform by using a paired-end read (v2, 2 × 250 bp) kit. The genome assemblies and annotations for all of the isolates were conducted by using the PATRIC v3.5.36 platform (11) and the Bacterial and Viral Bioinformatics Resource Center (BV-BRC) (12) with the default parameters, unless otherwise noted. A FastQC analysis (11) confirmed that the FastQ files were of good quality. The total number of reads was 2,688,201. We used the Comprehensive Genome Analysis Service, including *de novo* assembly by SPAdes (13), for the genome assembly of these isolates. Remapping the reads to the *de novo* assembly resulted in a median base coverage for Mycobacterium 189-2020, 539-2021, and 540-2021 (Table S1). The genome annotation was conducted by using the Rapid Annotations using Subsystems Technologies tool kit (RASTtk) (14). The annotated genome features are presented in Table S1. The Mycobacterium 189-2020, 539-2021, and 540-2021 genomes contained genes that were annotated as virulence factors (Virulence Factor Database [VFDB]), such as the FtsK/SpoIIIE family protein EccCa1, which is a component of the Type VII secretion system ESX-1, the ESAT-6-like protein EsxG, as well as antibiotic-resistance genes (Comprehensive Antibiotic Resistance Database [CARD]), such as the DNA gyrase subunit B, DNA gyrase subunit A, and transcriptional regulatory protein EmbR. The complete list of VFDB virulence factors and CARD antibiotic resistance genes for the isolates 189-2020, 539-2021, and 540-2021 is presented in Table S1. To identify the closest homologue in the database, as well as the closest high-quality representative genome in NCBI, we utilized the Similar Genome Finder Service, which uses the Mash/MinHash algorithm (15). Taxonomic classification via Kraken (16) identified isolates 189-2020, 539-2021, and 540-2021 as *M. pseudoshottsii*.

**FIG 2:**
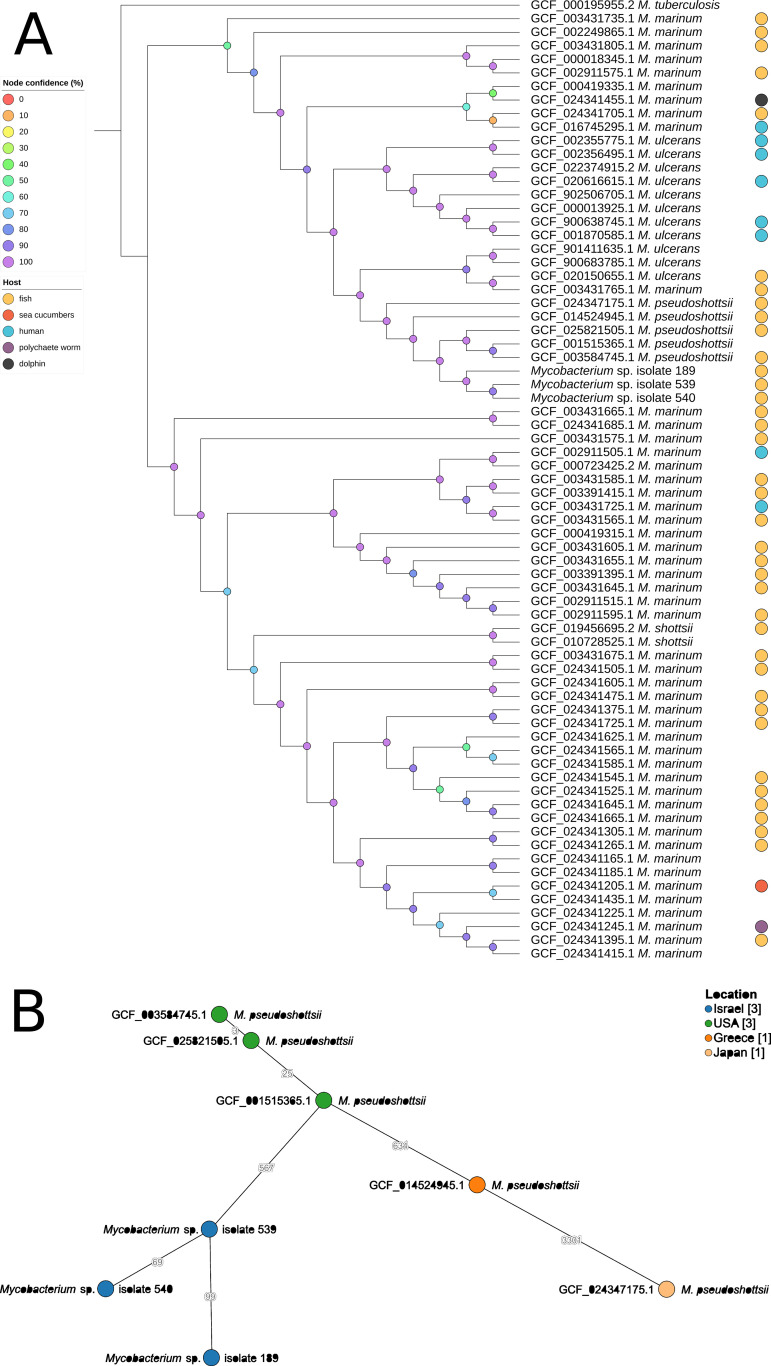
Phylogenetic analysis. (A) Maximum likelihood phylogenetic tree of 71 Mycobacterium ulcerans/Mycobacterium marinum clade (MuMC) isolates. The tree is based on a nucleotide alignment of 1,435 single-copy orthologous genes. The bootstrap values are shown for the internal nodes. (B) Minimum spanning tree of *M. pseudoshottsii* and our isolates, based on the alignment of 4,393 SNPs. The numbers on the edges represent the SNP differences between adjacent isolates. The difference between isolate 189-2020 and isolate 540-2021 is 82 SNPs (data not shown).

For the maximum likelihood phylogenetic tree construction (Fig. 2A), we used the Mycobacterium samples 189-2020, 539-2021, and 540-2021 with all of the available assemblies of M. marinum (excluding GCF_003431775.1), M. ulcerans, *M. shottsii*, and *M. pseudoshottsii* (Table S2) from the NCBI RefSeq database (17). In addition, M. tuberculosis H37Rv (accession number: GCF_000195955.2) was used to root the phylogenetic tree. To exclude inconsistencies between annotations, we used PGAP (18) to annotate our samples. OrthoFinder v2.5.4 (19) was used to perform orthogroup clustering between protein sequences, and this resulted in a set of 1,435 single-copy orthologous genes. For each orthogroup, corresponding coding sequences were aligned using Muscle v5.1 (20). The aligned sequences were concatenated and then analyzed with IQtree v1.6.12 (21) by using the GTR+F+I+G4 model that was selected by ModelFinder (22) with 1,000 ultrafast bootstrap replicates (23). The phylogenetic tree was visualized by using iTOL (24). kSNP v4.0 (25) was used to generate a total genomic single-nucleotide polymorphism (SNP) alignment for the minimum spanning tree of *M. pseudoshottsii* and our isolates (Fig. 2B). The tree was constructed using GrapeTree v1.5.0 (26). From the results of the minimum spanning tree, we cannot conclude that there is a clear epidemiological relationships between our isolates and isolates from other countries. Further analysis on a larger number of isolates is required in order to provide us with new insights.

The number of fish infections with *M. pseudoshottsii* has been increasing in recent years, and the reasons for this can be varied (increased awareness, improved surveillance systems, and availability of advanced diagnostic methods). The domestication of wild fish species, the intensification of modern aquaculture, and the international trade of live animals are also leading to higher numbers of reported cases of fish mycobacteriosis due to *M. pseudoshottsii*.

### Data availability.

The raw sequencing data generated from clinical fish samples have been deposited into the NCBI Sequence Read Archive (SRA) database under BioProject accession number PRJNA893417.

## References

[1] Fedrizzi T, Meehan CJ, Grottola A, Giacobazzi E, Fregni Serpini G, Tagliazucchi S, Fabio A, Bettua C, Bertorelli R, De Sanctis V, Rumpianesi F, Pecorari M, Jousson O, Tortoli E, Segata N. 2017. Genomic characterization of nontuberculous Mycobacteria. Sci Rep 7:45258. doi:10.1038/srep45258. 28345639 PMC5366915

[2] Tai AYC, Athan E, Friedman ND, Hughes A, Walton A, O'Brien DP. 2018. Increased severity and spread of *Mycobacterium ulcerans*, Southeastern Australia. Emerg Infect Dis 24:58–64. doi:10.3201/eid2401.171070. 28980523 PMC5749465

[3] Davidovich N, Pretto T, Sharon G, Zilberg D, Blum SE, Baider Z, Edery N, Morick D, Grossman R, Kaidar-Shwartz H, Dveyrin Z, Rorman E. 2020. Cutaneous appearance of mycobacteriosis caused by *Mycobacterium marinum*, affecting gilthead seabream (*Sparus aurata*) cultured in recirculating aquaculture systems. Aquaculture 528:735507. doi:10.1016/j.aquaculture.2020.735507.

[4] Stine CB, Jacobs JM, Rhodes MR, Overton A, Fast M, Baya AM. 2009. Expanded range and new host species of *Mycobacterium shottsii* and *M. pseudoshottsii*. J Aquat Anim Health 21:179–183. and doi:10.1577/H09-005.1. 20043404

[5] Jacobs J, Howard D, Rhodes M, May E, Harrell R, Harrell RM. 2009. Historical presence (1975–1985) of mycobacteriosis in Chesapeake Bay striped bass *Morone saxatilis*. Dis Aquat Organ 85:181–186. and doi:10.3354/dao02081. 19750805

[6] Matsumoto M, MacHida Y, Kanemaru M, Yamamoto M, Sano M, Kato G. 2022. Infection with *Mycobacterium pseudoshottsii* in cultured yellowtail *Seriola quinqueradiata* in Owase Bay, Japan. Fish Pathol 57:35–40. and doi:10.3147/jsfp.57.35.

[7] Mugetti D, Varello K, Gustinelli A, Pastorino P, Menconi V, Florio D, Fioravanti ML, Bozzetta E, Zoppi S, Dondo A, Prearo M. 2020. *Mycobacterium pseudoshottsii* in mediterranean fish farms: new trouble for european aquaculture? Pathogens 9:610–611. doi:10.3390/pathogens9080610. 32726963 PMC7459456

[8] Israeli Veterinary Services. Pre-marketing control of locally grown edible fish: procedure. 2017. Online]. Available https://www.gov.il/he/departments/policies/moag-pro-082.

[9] Kent KGP. 1985. Public health mycobacteriology. A guide for the level III laboratory. Atlanta, Georgia: US Department of Health and Human Services, Centers for Disease Control. 96–103.

[10] Gauthier DT, Haines AN, Vogelbein WK. 2021. Elevated temperature inhibits Mycobacterium shottsii infection and *Mycobacterium pseudoshottsii* disease in striped bass Morone saxatilis. Dis Aquat Organ 144:159–174. and doi:10.3354/dao03584. 33955854

[11] Wattam AR, Davis JJ, Assaf R, Boisvert S, Brettin T, Bun C, Conrad N, Dietrich EM, Disz T, Gabbard JL, Gerdes S, Henry CS, Kenyon RW, Machi D, Mao C, Nordberg EK, Olsen GJ, Murphy-Olson DE, Olson R, Overbeek R, Parrello B, Pusch GD, Shukla M, Vonstein V, Warren A, Xia F, Yoo H, Stevens RL. 2017. Improvements to PATRIC, the all-bacterial bioinformatics database and analysis resource center. Nucleic Acids Res 45:D535–D542. doi:10.1093/nar/gkw1017. 27899627 PMC5210524

[12] Olson RD. 2022. Introducing the Bacterial and Viral Bioinformatics Resource Center (BV-BRC): a resource combining PATRIC, IRD and ViPR. Nucleic Acids Res: 1–12. doi:10.1093/nar/gkac1003. 36350631 PMC9825582

[13] Bankevich A, Nurk S, Antipov D, Gurevich AA, Dvorkin M, Kulikov AS, Lesin VM, Nikolenko SI, Pham S, Prjibelski AD, Pyshkin AV, Sirotkin AV, Vyahhi N, Tesler G, Alekseyev MA, Pevzner PA. 2012. SPAdes: a new genome assembly algorithm and its applications to single-cell sequencing. J Comput Biol 19:455–477. doi:10.1089/cmb.2012.0021. 22506599 PMC3342519

[14] Brettin T, Davis JJ, Disz T, Edwards RA, Gerdes S, Olsen GJ, Olson R, Overbeek R, Parrello B, Pusch GD, Shukla M, Thomason JA, Stevens R, Vonstein V, Wattam AR, Xia F. 2015. RASTtk: a modular and extensible implementation of the RAST algorithm for building custom annotation pipelines and annotating batches of genomes. Sci Rep 5. doi:10.1038/srep08365. PMC4322359 25666585

[15] Ondov BD, Treangen TJ, Melsted P, Mallonee AB, Bergman NH, Koren S, Phillippy AM. 2016. Mash: fast genome and metagenome distance estimation using MinHash. Genome Biol 17:1–14. doi:10.1186/s13059-016-0997-x. 27323842 PMC4915045

[16] Wood DE, Salzberg SL. 2014. Kraken: ultrafast metagenomic sequence classification using exact alignments. Genome Biol 15. doi:10.1186/gb-2014-15-3-r46. PMC4053813 24580807

[17] O’Leary NA, et al. 2016. Reference sequence (RefSeq) database at NCBI: current status, taxonomic expansion, and functional annotation. Nucleic Acids Res 44:D733–D745. doi:10.1093/nar/gkv1189. 26553804 PMC4702849

[18] Li W, O'Neill KR, Haft DH, DiCuccio M, Chetvernin V, Badretdin A, Coulouris G, Chitsaz F, Derbyshire MK, Durkin AS, Gonzales NR, Gwadz M, Lanczycki CJ, Song JS, Thanki N, Wang J, Yamashita RA, Yang M, Zheng C, Marchler-Bauer A, Thibaud-Nissen F. 2021. RefSeq: expanding the Prokaryotic Genome Annotation Pipeline reach with protein family model curation. Nucleic Acids Res 49:D1020–D1028. doi:10.1093/nar/gkaa1105. 33270901 PMC7779008

[19] Emms DM, Kelly S. 2019. OrthoFinder: phylogenetic orthology inference for comparative genomics. Genome Biol 20:1–14. doi:10.1186/s13059-019-1832-y. 31727128 PMC6857279

[20] Edgar RC. 2004. MUSCLE: multiple sequence alignment with high accuracy and high throughput. Nucleic Acids Res 32:1792–1797. doi:10.1093/nar/gkh340. 15034147 PMC390337

[21] Nguyen LT, Schmidt HA, Von Haeseler A, Minh BQ. 2015. IQ-TREE: a fast and effective stochastic algorithm for estimating maximum-likelihood phylogenies. Mol Biol Evol 32:268–274. and doi:10.1093/molbev/msu300. 25371430 PMC4271533

[22] Kalyaanamoorthy S, Minh BQ, Wong TKF, Von Haeseler A, Jermiin LS. 2017. ModelFinder: fast model selection for accurate phylogenetic estimates. Nat Methods 14:587–589. and doi:10.1038/nmeth.4285. 28481363 PMC5453245

[23] Hoang DT, Chernomor O, von Haeseler A, Minh BQ, Vinh LS. 2018. UFBoot2: improving the ultrafast bootstrap approximation. Mol Biol Evol 35:518–522. and doi:10.1093/molbev/msx281. 29077904 PMC5850222

[24] Letunic I, Bork P. 2021. Interactive tree of life (iTOL) v5: an online tool for phylogenetic tree display and annotation. Nucleic Acids Res 49:W293–W296. doi:10.1093/nar/gkab301. 33885785 PMC8265157

[25] Gardner SN, Slezak T, Hall BG. 2015. kSNP3.0: SNP detection and phylogenetic analysis of genomes without genome alignment or reference genome. Bioinformatics 31:2877–2878. and doi:10.1093/bioinformatics/btv271. 25913206

[26] Zhou Z, Alikhan N-F, Sergeant MJ, Luhmann N, Vaz C, Francisco AP, Carriço JA, Achtman M. 2018. Grapetree: visualization of core genomic relationships among 100,000 bacterial pathogens. Genome Res 28:1395–1404. doi:10.1101/gr.232397.117. 30049790 PMC6120633

